# Influence of the circadian cycle, sex and production stage on the reference values of parameters related to stress and pathology in porcine saliva

**DOI:** 10.1186/s40813-023-00337-7

**Published:** 2023-09-29

**Authors:** Y. Saco, R. Peña, M. Matas-Quintanilla, F. J. Ibáñez-López, M. Piñeiro, J. Sotillo, A. Bassols, A. M. Gutiérrez

**Affiliations:** 1 Departament de Bioquímica i Biologia Molecular i Servei de Bioquímica Clínica Veterinària, Facultat de Veterinària, Universitat Autònoma de Barcelona, Bellaterra, 08193 Spain; 2 BioVetMed Research Group, Department of Animal Medicine and Surgery, Veterinary School, CEIR Campus Mare Nostrum (CMN), University of Murcia, Murcia, 30100 Spain; 3 Department of Didactic of Maths and Social Sciences, CEIR Campus Mare Nostrum (CMN), University of Murcia, Murcia, 30008 Spain; 4 Acuvet Biotech, C/Bari 25, Zaragoza, 50197 Spain

**Keywords:** Saliva, Biomarker, Circadian rhythm, Reference intervals, Acute phase proteins, S100A12, ADA, Cu, Zn, Oxidative status

## Abstract

**Background:**

The concentration of biomarkers in saliva could be influenced by several factors not related to the specific condition under analyses, which should be considered for proper clinical interpretation. In the present study, the circadian rhythm of C-reactive protein (CRP), haptoglobin (Hp), Pig-MAP, S100A12, Cu, Zn, Adenosine deaminase (ADA), total protein (TP), total antioxidant capacity (TAC), total oxidant status (TOS), oxidative stress index (OSI), cortisol and α-amylase in saliva of 20 female and 20 male pigs was investigated. Moreover, the influence of sex and production phase (post-weaning, fattening and finishing) on the concentrations of biomarkers in a total of 414 healthy pigs was studied and the reference intervals for all salivary biomarkers were calculated accordingly.

**Results:**

All parameters except Pig-MAP, OSI and α-amylase varied significantly along the daytime, and most of them peak around early afternoon (13–15 h). The cosinor analysis described the temporal dynamics of circadian rhythms for all parameters. The range values showed differences between male and female pigs in 8 out of the 13 biomarkers, with higher concentrations in females in comparison to male pigs. The influence of the production phase on the salivary concentrations was observed for all the biomarkers. The highest concentrations were observed for Pig-MAP, S100A12 and α-amylase in post-weaning animals, for TP in growing pigs and for OSI in finishing animals. Most of the sex-influenced biomarkers showed the highest concentrations at growing stages with some exceptions such as ADA or Hp that showed the peak at finishing and post-weaning stages respectively.

**Conclusions:**

It is necessary to establish the optimal daytime for routine saliva sampling to avoid circadian variations and for that end, the time interval between 10:00 a.m. to 12:00 a.m. is highly recommended. The factors sex and production phase influence the concentration of biomarkers and should be considered for proper biomarker interpretation. The reference intervals presented here for each salivary biomarker will help to correctly interpret the results of these analytes and contribute to the use of saliva as a non-invasive sample for the diagnosis and monitoring of the health status of swine farms.

**Supplementary Information:**

The online version contains supplementary material available at 10.1186/s40813-023-00337-7.

## Background

Over the years, saliva has become an important sample to assess the health status in pigs, due to its non-invasive character and easy collection that does not need experienced staff. Components of saliva mirror in many cases those from plasma and they may provide a good indication of the health status of the individual [1, 2]. Moreover, saliva shows proteins originated from the different salivary sources that could also be of interest for health assessment and monitoring [1]. Several compounds related to stress, oxidation, inflammation, immune activation and other causes have already been assayed in pig saliva. One of the first analytes to be measured was the stress hormone cortisol since the collection of saliva may be performed without altering the animal, being a more adequate indicator of stress compared to the plasma hormone [3]. Furthermore, it is known that only free cortisol is found in saliva and that there is an equilibrium between the concentration of cortisol in saliva and free cortisol in plasma, although concentration in saliva is much lower than in plasma (5–10% of the free plasma cortisol concentration) [4]. Besides cortisol, many other analytes related to stress and pathology have been determined in pig saliva including acute phase proteins (APPs) [5–7], α-amylase [8, 9], carbonic anhydrase VI [10], interleukin (IL)-18 [11], immunoglobulins (Ig) A, G and M [12, 13], adenosine deaminase (ADA) [14], chromogranin A [15], markers of the oxidative status [16] and trace elements [17].

Variations in the pig saliva concentration of one or more of these parameters are indicative of stress [2, 11, 12, 18–22] and several pathologies [2, 6, 14, 16, 23, 24], increasing the interest on these biomarkers and this type of sample for the diagnosis and monitoring of the health status of swine farms.

On this regard, it is of the uttermost importance to analyze in detail all the potential sources of results variation, due to the conditions of the individual and/or differences in the preanalytical phase of the procedure. It has to be considered that the saliva flux is variable, in contrast to the plasma volume, and it may be affected by the circadian cycle. Likewise, it is essential to establish reference intervals (RI) not only for each animal species, but also taking into account variables like age and management conditions. In fact, the effect of age, breed and productive stage has been already described by our group for salivary ADA, total antioxidant capacity (TAC) and the APPs haptoglobin (Hp) and C-reactive protein (CRP) [16], as well as the influence of the circadian rhythm on salivary cortisol [25, 26] and Hp and CRP [27].

Thus, the objective of the present study is to define the optimal daytime for routine saliva sampling and to establish actual reference range values for salivary biomarkers of health and welfare status in commercial pigs, specifically for CRP, Hp, Pig-MAP, S100A12, Cu, Zn, ADA, total protein (TP), TAC, total oxidant status (TOS), oxidative stress index (OSI), cortisol and α-amylase.

## Results

### Circadian rhythm trial

**Fig. 1:**
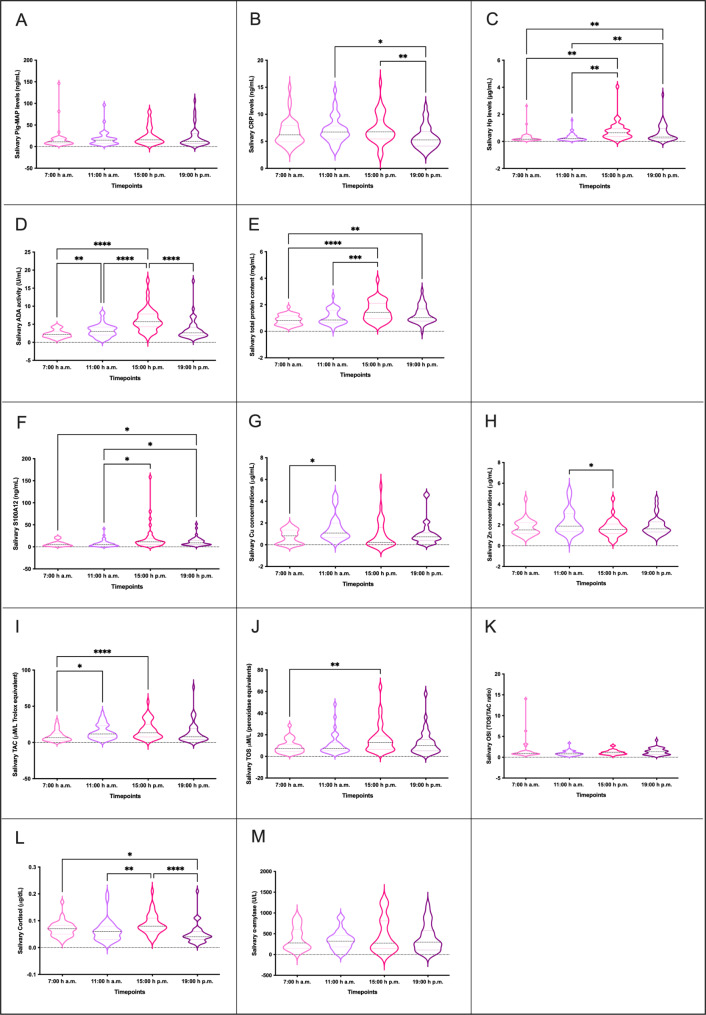
Concentration of salivary analytes studied at different timepoints in healthy pigs. Pig-MAP (ng/mL) **(A)**, CRP (ng/mL) **(B)**, Hp (µg/mL) **(C)**, ADA (U/mL) **(D)**, TP (mg/mL) **(E)**, S100A12 (µg/mL) **(F)**, Cu (µg/mL) **(G)**, Zn (µg/mL) **(H)**, TAC (µM/L Trolox equivalents) **(I)**, TOS (µM/L peroxidase equivalents) **(J)**, OSI (TOS/TAC ratio) **(K)**, cortisol (µg/dL) **(L)** and salivary α-amylase (U/L) **(M)** concentrations at different timepoints (7:00 a.m., 11:00 a.m., 15:00 p.m. and 19 p.m.). Graph showing the distribution of the population, the median (central horizontal line), 25th and 75th percentiles (non-central horizontal lines within the plot), maximum and minimum (edges of the figure). Statistical differences are indicated by *, **, ***, and **** for p < 0.05, p < 0.01, p < 0.001, and p < 0.0001, respectively

Statistically significant differences in the concentrations of salivary biomarkers during the daytime were observed for all parameters except Pig-MAP, OSI and α-amylase (Fig. 1). The highest values of biomarkers were reported in the saliva samples collected at 15:00 p.m. except for trace elements in which the highest values were reported at 11:00 a.m. No differences were observed in the daytime variations of salivary parameters between male and female (*p* value > 0.05 for interaction between sex and time). The power of the differences found in the concentration of salivary parameters between timepoints were higher to 90% for all analyses (Table 1).

**Table 1:** Achieved power for the analysis of variations in the concentration of salivary biomarkers during the daytime and the effect size of the differences observed between timepoints

Salivary biomarkers^1^	Difference between timepoints^2^	P value	Power	Effect size^3^					
				t1-t2	t1-t3	t1-t4	t2-t3	t2-t4	t3-t4
Pig-MAP	No	0.265	90.28						
CRP	Yes	0.011	90.59					0.516	0.555
Hp	Yes	9.69e-05	90.41		0.632	0.557	0.650	0.622	
ADA	Yes	3.37e-11	95.41	0.540	1.32		0.963		0.895
TP	Yes	9.94e-07	91.77		0.986	0.633	0.657		
S100A12	Yes	0.013	90.26			0.426	0.435	0.467	
Cu	Yes	0.029	90.94	0.643					
Zn	Yes	0.029	90.96				0.397		
TAC	Yes	0.002	90.65	0.501	0.818				
TOS	Yes	0.011	90.98		0.655				
OSI	No	0.200	90.46						
Cortisol	Yes	5.19e-05	90.35			0.399	0.544		0.815
α-amylase	No	0.356	90.28						

^1^Salivary biomarkers measured in a total of 20 female and 20 male healthy pigs

^2^Timepoints analysed: 7:00 a.m., 11:00 a.m., 15:00 p.m. and 19 p.m.

^3^Effect size: Cohen’s D

**Fig. 2:**
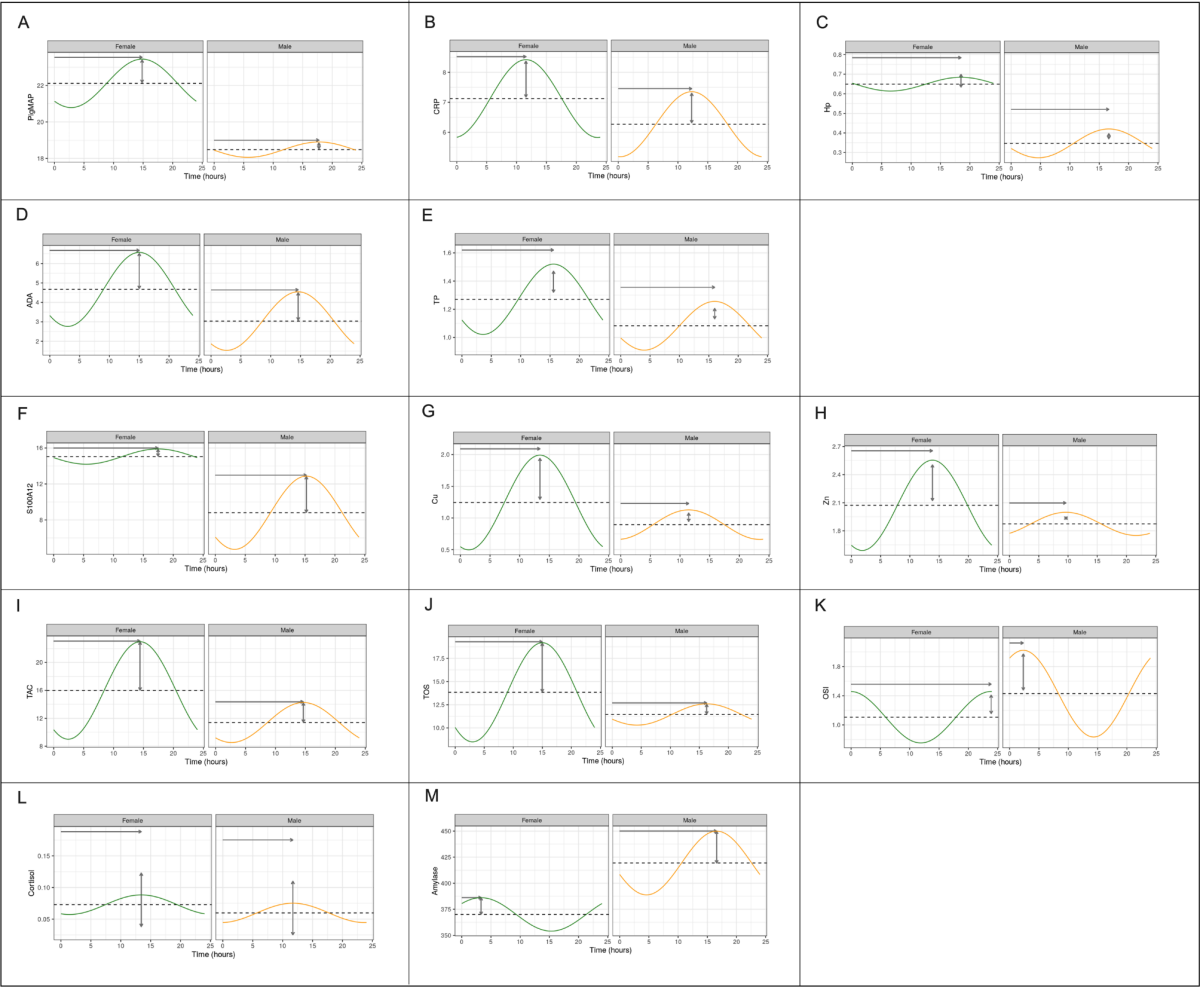
Fitted cosine curves of the different salivary analytes studied in female (n = 20) and male (n = 20) pigs in the circadian study in which animals were sampled at 4 h interval in a 12 h period between 7:00 a.m. and 19:00 p.m. Pig-MAP (ng/mL) **(A)**, CRP (ng/mL) **(B)**, Hp (µg/mL) **(C)**, ADA (U/mL) **(D)**, TP (mg/mL) **(E)**, S100A12 (µg/mL) **(F)**, Cu (µg/mL) **(G)**, Zn (µg/mL) **(H)**, TAC (µM/L Trolox equivalents) **(I)**, TOS (µM/L peroxidase equivalents) **(J)**, OSI (TOS/TAC ratio) **(K)**, cortisol (µg/dL) **(L)** and salivary α-amylase (U/L) **(M)**

The cosinor model showed the circadian curves for all parameters (Fig. 2). Overall, the concentration of salivary biomarkers peaks between 13:00–15:00 p.m. for most of the parameters. For OSI, the highest concentrations were obtained at midnight while α-amylase showed different peaks for male (17:00 p.m.) and females (3:00 a.m.). Statistically significant differences between the mean midline-estimating statistics of rhythm (MESOR), or acrophase by sex were observed for ADA and Hp (MESOR) and for OSI (acrophase) (Table 2).

**Table 2:** Extracted parameters from the cosinor method analysis in female (n = 20) and male (n = 20) pigs

Salivary parameter	MESOR		Amplitude		Acrophase	
	Mean	CI (2.5–97.5%)	Mean	CI (2.5–97.5%)	Mean	CI (2.5–97.5%)
Pig-MAP						
Female	22.11	15.27–29.16	1.32	-6.46–4.59	-3.84	-7.44 - -1.15
Male	18.48	12.01–25.31	0.42	-8.66–2.61	-4.64	-6.16 - -2.90
CRP						
Female	7.12	6.40–7.85	1.29	0.27–2.24	-3.02	-3.64 - -2.49
Male	6.26	5.56–7.01	1.08	0.11–2.06	-3.22	-4.16 - -2.34
Hp						
Female	0.64*	0.50–0.79	0.03	-0.16–0.11	-4.84	-5.59 - -4.06
Male	0.34	0.19–0.49	0.07	-0.13–0.19	-4.35	-5.35 - -3.21
ADA						
Female	4.66*	4.00–5.25	1.90	0.74–3.02	-3.92	-4.23 - -3.60
Male	3.03	2.40–3.69	1.50	0.42–2.55	-3.82	-4.20 - -3.42
TP						
Female	1.27	1.09–1.45	0.24	-0.02–0.49	-4.08	-4.57 - -3.52
Male	1.08	0.90–1.26	0.17	-0.05–0.37	-4.18	-4.77 - -3.50
S100A12						
Female	15.04	10.67–19.26	0.84	-6.00–3.28	-4.57	-5.75 - -3.35
Male	8.80	4.16–13.36	4.07	-2.54–9.58	-3.98	-5.11 - -2.78
Cu						
Female	1.24	0.93–1.56	0.74	0.23–1.23	-3.51	-3.91 - -3.05
Male	0.89	0.58–1.20	0.23	-0.18–0.56	-3.00	-5.54 - -0.97
Zn						
Female	2.07	1.80–2.32	0.48	-0.01–0.94	-3.62	-4.44 - -2.79
Male	1.87	1.61–2.12	0.12	-0.26–0.34	-2.52	-5.18 - -0.07
TAC						
Female	15.98	12.07–19.63	6.98	2.55–11.30	-3.77	-4.11 - -3.39
Male	11.39	7.45–15.03	2.86	-1.27–6.53	-3.83	-5.17 - -2.36
TOS						
Female	13.84	10.49–17.02	5.34	0.99–9.79	-3.92	-4.38 - -3.37
Male	11.45	8.42–14.46	1.14	-2.65–3.27	-4.25	-6.12 - -2.36
OSI						
Female	1.10	0.80–1.44	0.35	-0.18–0.83	-6.25*	-14.09 - -4.24
Male	1.42	1.10–1.74	0.59	0.01–1.17	-0.60	-2.10–1.28
Cortisol						
Female	0.07	0.06–0.08	0.01	-0.00–0.03	-3.51	-4.60 - -2.35
Male	0.05	0.05–0.06	0.01	-0.00–0.03	-3.06	-4.30 - -2.02
α-amylase						
Female	370.06	280.09–458.33	16.03	-114.37–58.01	-0.86	-3.10–4.35
Male	419.35	327.95–512.80	30.58	-100.36–97.99	-4.34	-6.12 - -2.45

*Statistically significant differences between female and male parameters (*p* < 0.05)

### Reference interval trial

The concentrations of 5 biomarkers (Pig-MAP, S100A12, α-amylase, TP and OSI) were significantly different in pigs at different production stages but similar in male and female pigs, while the other 8 biomarkers showed statistically significant differences in both factors (production stage and sex).

**Fig. 3:**
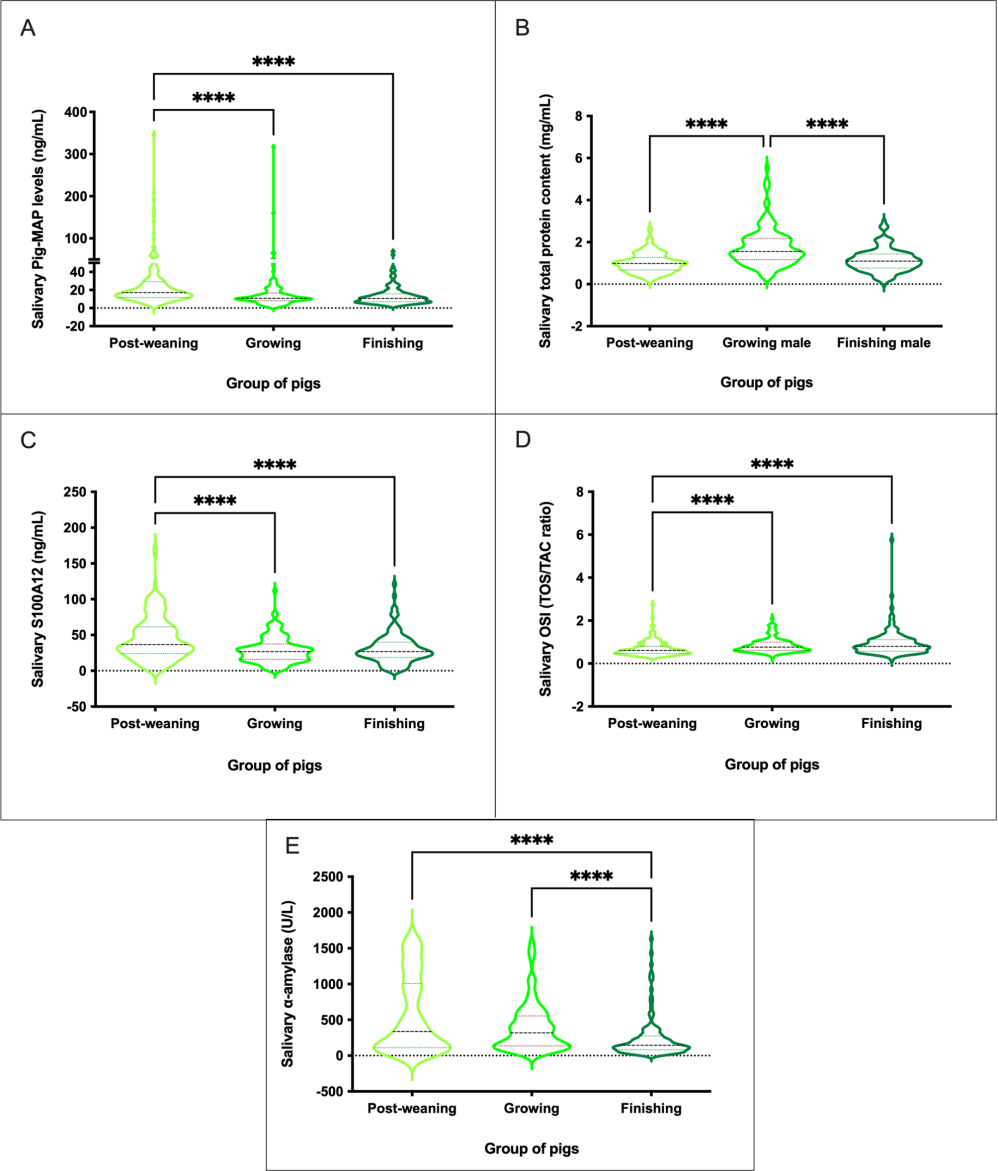
Concentration of salivary analytes in pigs at different stages of the production system. Pig-MAP (ng/mL) **(A)**, TP (mg/mL) **(B)**, S100A12 (µg/mL) **(C)**, OSI (TOS/TAC ratio) **(D)** and salivary α-amylase (U/L) **(E)**. Graph showing the distribution of the population, the median (central horizontal line), 25th and 75th percentiles (non-central horizontal lines within the plot), maximum and minimum (edges of the figure). Statistical differences are indicated by **** for p < 0.0001

Three RIs were established for those biomarkers that showed an influence of production phase in the salivary levels (Table 3), specifically RI for post-weaning, growing, and finishing pigs. The highest levels of salivary biomarkers were observed in post-weaning animals for Pig-MAP, S100A12 and α-amylase, in growing pigs for TP and in finishing animals for OSI (Fig. 3).

**Table 3:** Reference range intervals in commercial pigs at different production stages for salivary biomarkers not sex-influenced

Salivary biomarker^1^	Reference Interval	Lower and Upper Confidence Interval	N
Pig-MAP			
Post-weaning	7.07–100.64	4.44–7.19; 98.69–207.72	156
Growing	4.37–29.30	3.43–5.24; 26.01–33.92	117
Finishing	3.65–36.79	2.72–3.74; 35.56–68.73	124
S100A12			
Post-weaning	7.75–99.77	1.76–7.92; 100.24 – NA^2^	156
Growing	3.59–64.32	4.67–3.99; 64.58 – NA^2^	130
Finishing	6.61–60.68	3.24–8.38; 44.71–68.87	115
TP			
Post-weaning	0.36–1.78	0.21–0.37; 1.78–2.30	157
Growing	0.76–3.20	0.43–0.78; 3.19–4.74	126
Finishing	0.31–2.08	0.14–0.37; 2.03–2.39	119
OSI			
Post-weaning	0.32–1.37	0.27–0.33; 1.77 – NA^2^	160
Growing	0.49–1.61	0.42–0.49; 2.09 – NA^2^	130
Finishing	0.42–1.88	0.34–0.44; 2.24 – NA^2^	122
α-amylase			
Post-weaning	36.19–1576.61	19.0–36.03; 1577.24–1685.32	160
Growing	57.90–1174.28	28.82–58.95; 1079.44–1579.21	131
Finishing	27.83–1027.53	22.27–28.82; 918.97–1632.92	124

^1^Pig-MAP (ng/mL); S100A12 (µg/mL); TP (mg/mL); OSI (Ratio TOS/TAC); α-amylase (U/L).

^2^NA = not available

**Fig. 4:**
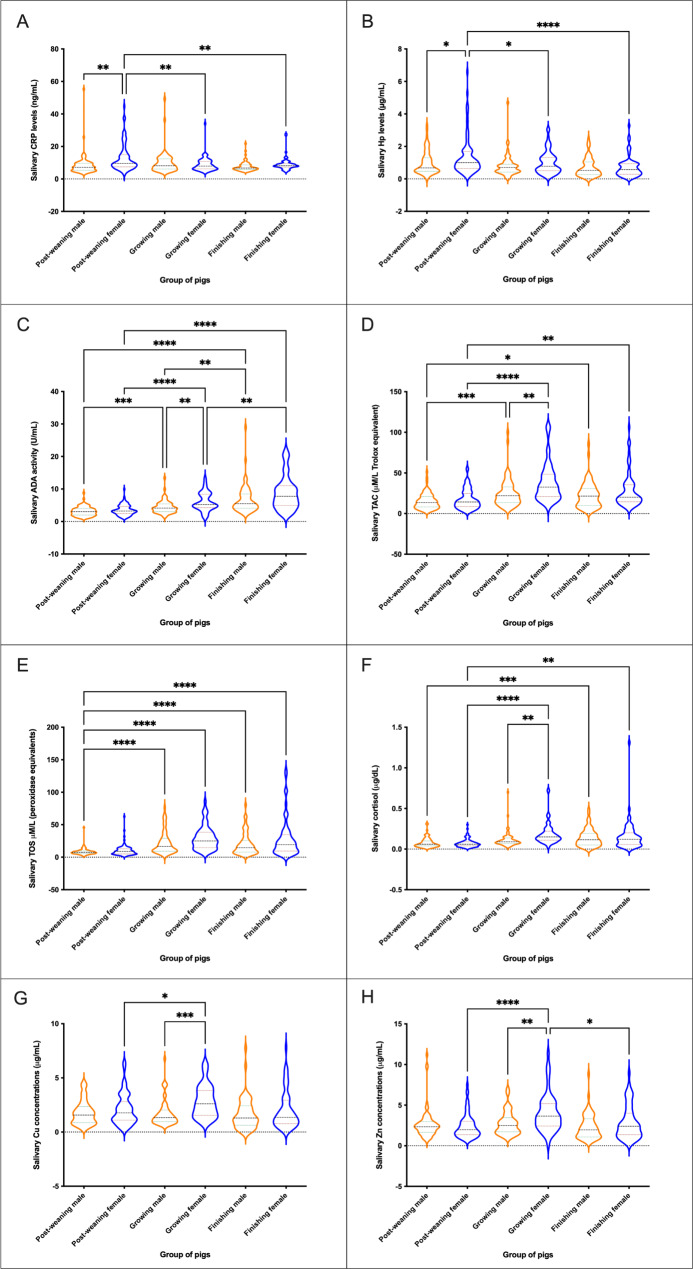
Concentration of salivary analytes in pigs of different sexes and stages of the production system. CRP (ng/mL) **(A)**, Hp (µg/mL) **(B)**, ADA (U/mL) **(C)**, TAC (µM/L Trolox equivalents **(D)**, TOS (µM/L peroxidase equivalents) **(E)**, cortisol (µg/dL) **(F)**, Cu (µg/mL) **(G)**, and Zn (µg/mL) **(H)**. Graph showing the distribution of the population, the median (central horizontal line), 25th and 75th percentiles (non-central horizontal lines within the plot), maximum and minimum (edges of the figure). Statistical differences are indicated by *, **, ***, and **** for p < 0.05, p < 0.01, p < 0.001, and p < 0.0001, respectively

For the biomarkers that showed an influence of sex and production stage in their salivary concentrations, six RIs were defined (Table 4). Overall, for all salivary biomarkers higher concentrations were recorded in female pigs in comparison to male counterparts. Moreover, the concentration of most biomarkers was higher at growing stages in males and females, except for ADA that showed the highest values at finishing and Hp and CRP that showed highest values at growing in males while the highest values were recorded at post-weaning state in females (Fig. 4).

**Table 4:** Reference range intervals in male and female pigs at different production stages for salivary biomarkers

Salivary biomarker^1^	Male pigs			Female pigs		
	Reference interval	Confidence interval	N	Reference interval	Confidence interval	N
CRP						
Post-weaning	3.99–14.39	3.50–4.59; 10.79–17.13	79	5.53–29.60	5.13–6.13; 20.79–33.79	79
Growing	4.47–16.20	3.88–4.81; -13.62–18.24	64	4.62–15.00	3.85–5.10; 2.57–17.02	67
Finishing	4.69–14.56	4.46–4.85; 7.82–18.96	62	6.25–12.97	5.84–6.80; 12.24–14.95	53
Hp						
Post-weaning	0.28–2.40	0.21–0.43; 1.72–2.70	79	0.31–4.35	0.19–0.40; 3.36–5.69	79
Growing	0.20–1.43	0.08–0.31; 1.09–1.57	59	0.20–2.44	0.12–0.30; 1.84–2.88	67
Finishing	0.07–2.14	0.00–0.08; 1.82–2.78	62	0.09–2.35	-0.01–0.11; 1.49–3.17	61
ADA						
Post-weaning	1.23–5.39	1.14–1.35; 3.38–5.77	80	1.32–6.26	1.07–1.59; 5.10–6.81	80
Growing	1.96–7.92	1.30–2.45; 3.31–8.94	64	1.60–11.60	0.36–2.12; 9.94–13.46	67
Finishing	2.81–13.37	2.46–3.35; 0.10–16.04	62	2.20–20.53	1.33–2.58; 19.20–24.38	62
Cu						
Post-weaning	0.46–3.52	0.33–0.61; 3.38–4.18	64	0.34–4.29	0.09–0.42; 3.92–5.02	71
Growing	0.69–2.94	0.63–0.82; 2.49–3.56	51	0.82–4.96	0.5–0.9; 4.22–5.59	54
Finishing	0.00–3.32	-0.19–0.01; 3.11–3.57	54	0.27–4.70	0.12–0.35; 4.28–5.75	53
Zn						
Post-weaning	0.93–4.44	0.57–1 02; 3.84–5.20	76	0.77–5.89	0 56–0.85; 4.77–7.12	80
Growing	1.01–6.44	0.65–1.07; 5.85–8.15	64	1.35–9.08	1.01–1.86; 9.06–11.06	65
Finishing	0.60–4.96	0.36–0.71; 1.10–5.83	62	0.65–6.74	0.53–0.93; 4.54–7.23	62
TAC						
Post-weaning	3.57–31.59	2.39–3.93; 19.11–35.14	80	3.29–40.75	1.17–3.75; 27.18–47.07	80
Growing	6.83–51.87	5.26–8.45; 6.49–58.33	64	13.50–102.94	11.90–18.00; 99.36–133.69	67
Finishing	4.02–47.36	1.67–5.48; 9.86–54.00	62	8.83–85.30	6.83–11.56; 67.10–108.17	62
TOS						
Post-weaning	3.94–15.57	3.26–4.46; 13.63–17.84	77	2.79–21.17	1.61–3.62; 10 01–26.21	79
Growing	5.02–62.49	2.38–7.21; 51.37–73.97	64	8.39–70.01	7.02–10.69; 55.31–84.51	67
Finishing	3.88–61.68	2.45–5.72; 43.82–78.64	62	3.43–102.52	1.04–4.13; 72.30–139.37	62
Cortisol						
Post-weaning	0.01–0.17	0.01–0.02; 0.16–0.20	75	0.01–0.15	0.00–0.02; 0.14–0.18	73
Growing	0.03–0.21	0.01–0.03; 0.17–0.27	62	0.07–0.37	0.06–0.11; 0.33–0.45	65
Finishing	0.01–0.28	-0.00–0.02; 0.23–0.31	58	0.01–0.37	-0.01–0.01; 0.26–0.45	61
^1^CRP (ng/mL); Hp (𝛍g/mL); ADA (U/mL); Cu (𝛍g/mL); Zn (𝛍g/mL); TAC (µM Trolox equivalents/L); TOS (µM Peroxidase equivalents/L); Cortisol (𝛍g/dL).						

## Discussion

To use saliva as a routine sample type to assess pig health it is mandatory to acknowledge and characterize the potential sources of variation that may affect the analytical results, like it is well accepted for plasma parameters [28]. Furthermore, it is necessary to extend the experimental studies on the calculation of RIs to provide a robust set of data to be used by the clinician.

Amongst the sources of variation, the influence of the circadian rhythm is important, as demonstrated by the well-known example of salivary cortisol [25, 26]. To describe the circadian rhythms of the different tested parameters, we have used the cosinor method described previously by Ekkel et al. to assess the circadian rhythm of salivary cortisol in pigs [18, 25]. In this method, point and 95% confidence interval estimates of the following rhythm characteristics are obtained: the MESOR (the rhythm-adjusted mean), the circadian amplitudes (measures of the extent of the predictable change within one cycle), and the circadian acrophases (measures of the timing of overall high values recurring in each cycle). Using this chronobiological approach, not only the average cortisol level is found but the amplitude of the circadian rhythm is also analysed [25, 29, 30]. In the present study, the salivary cortisol rhythmicity is similar to other reports, peaking around noon or early afternoon [31–33]. Pigs in the present study were 120 days old and were sampled in spring. Although the circadian rhythms may be influenced due to environmental conditions [31], gender or age [32] being less marked in very young animals, it has been described that around 20 weeks of age they reach an adult and stable profile [32]. The similarities between the fitted curve of our 12-hour study, 4-hourly values, and the one reported before for 24-hours covering [32], showed the usefulness of cosinor model for rhythmicity parameters comparison when sampling frequency are restricted to few times along the scale of a given rhythm as reported before [29].

Besides cortisol, the influence of the circadian rhythm on other salivary parameters has been studied by us in the present work. Thus, the influence on CRP was found to be significant with the lowest concentrations at 19:00 p.m., and a fitted cosinor curve similar to the one observed for cortisol in this study and in previous reports [34] with peak concentrations in the late morning. Hp is higher in the afternoon than in the morning, with no additional variations between males and females but with higher MESOR values in females than in males. This difference in the MESOR between males and females was also observed for ADA concentrations and could be explained by the sex influence in the levels of salivary biomarkers as reported before [33, 35]. Moreover, our peak around 15:00 p.m. in ADA levels also agree with the results reported for the peaks observed in ADA isoforms [33]. Although Pig-MAP showed no variations in the mean concentrations during the daytime, the cosinor analysis revealed a circadian curve with higher concentrations in the early afternoon. Regarding the other parameters, the majority of them (TP, S100A12, Cu, Zn, TAC and TOS) are affected by the daytime except OSI and α-amylase which are not affected. However, the cosinor curves indicated that most of the parameters peak around early afternoon (13:00-15:00 p.m.) with the exception of Cu and Zn in males (peak between 10:00 a.m. and 11:00 a.m.). The curves of females and males behave similarly for most of the parameters studied, although the amplitude, which measure the extent of rhythmic change, is clearly higher in females for Pig-MAP, Cu, Zn, TAC and TOS, whereas the amplitude is clearly higher in males for S100A12. The curves for OSI and α-amylase are different in shape between females and males, showing different peaks for the concentrations in females and males. Our results in male pigs agree with previous studies in which a peak of α-amylase was observed at around 16:00 p.m. [33]. However, we observed differences in the TAC levels over the daytime peaking at 15:00 p.m., with a statistical power of 90.95%, which is contradictory to the results of the latest study. It has been described that circadian rhythms have a significant impact on oxidative stress, but there is still an incomplete understanding of the molecular mechanisms linking circadian rhythms and oxidative stress [36] that should be further studied.

Nevertheless, the variations observed in all parameters fall within the normal range and they would not have any clinical significance as reported before for APPs [27].

It is difficult to compare these results to other animal species since there is very little or no information about the influence of the sampling time in salivary biomarker concentrations. In humans, the influence of daytime was analysed for salivary α-amylase which peaks in the afternoon (similar to our results in male pigs) [37], and for salivary antioxidant mechanisms which show several regular peaks along the daytime [38]. Our results show that TAC and TOS peak in early afternoon, but it is difficult to compare the results since the analytical techniques were different.

In general, it is clear that daytime may affect the concentration of many components in saliva [34], as studies performed in humans have already shown for melatonin [39], androgen derivatives [40], some steroid hormones and IgA [41], peptide hormones such as oxytocin and arginine-vasopressin [42], serotonin [43] and minerals such as Ca, P, Na and K [44]. None of these compounds were assayed in the present work.

In other animal species, some research has been carried out in goats but referred only to electrolytes and urea and found diurnal acrophases for most of the tested parameters [45].

We recommend establishing a time interval for routine saliva sampling to avoid misinterpretations due to circadian variations. The optimal time interval should be compatible with farm and laboratory timetable to guarantee optimal biomarker analysis and/or sample storage, so the time interval between 10:00 a.m. and 12:00 a.m. is highly recommended.

Each of the salivary parameters studied has its own clinical significance and the information from one could not be replaced by other, but compatible. Those biomarkers without circadian influence in its levels, specifically Pig-MAP, α-amylase and OSI, would be preferable than others, however, for biomarker’s recommendation other factor should also be considered such as the specific conditions to be evaluated, since not all the conditions alter the level of biomarkers in a similar way [19, 63].

On the other hand, RIs must be set for each animal species, and the knowledge of the influence of age and management conditions has been recognized as an essential condition to the correct use of these RI [28, 46]. Several reports have analysed the effects of age, breed or management conditions on plasma/serum parameters including APPs and metabolic compounds [47–53] and found relevant differences in RI values depending on the conditions. Thus, the plasma antioxidant potential, hydroperoxides, the oxidative stress index (OSI) and vitamin A and E differed in the young piglets depending on the weaning status [47]. Sex and age have an influence of serum acute phase proteins [48, 50–54], and plasma cytokines [51, 52]. Many of the most important differences occurs in young ages and around weaning, but they occur also in adult ages and depending on the conditions of the farm [53].

Despite the amount of information on serum or plasma analytes, scarce information is available on saliva. Our group has previously described the influence of sex, breed (Iberian or Large White x Duroc) and the production phase (post-weaning, nursery, fattening, and finishing) on ADA, CRP, Hp and TAC [16]. The results presented in this work extend this information to other salivary parameters. However, our study is limited to commercial entire male and female pigs and additional studies should be performed to extent the analysis to castrated or even immunocastrated pigs. Moreover, our results are limited to some commercial standardized genetics and further studies should be performed to cover other hybrids of other genetic lines.

Our results indicate that significant differences exist in several parameters depending on the production stage but were not affected by sex: Pig-MAP, S100A12 and α-amylase present higher values in post-weaning animals, whereas TP is higher in growing pigs and OSI in finishing pigs. Furthermore, on the other tested parameters, female animals showed higher RIs than males, and most of them higher values in the growing phase except for ADA, Hp and CRP. Our results are in concordance with previous ones in which higher levels of immune markers have been reported in saliva samples of female in comparison to age-matched male pigs [35]. Regarding CRP, Hp, ADA and TAC, our results were also similar to our previous study [16] taking into account the technical and biological differences between the animals included in each study.

The interest of the results described here on the influence of the sampling time and the calculation of reference intervals, using a high number of individuals, resides on the previously reported study by us showing that these parameters measured in saliva may provide a diagnostic tool more adequate than serum to evaluate the health status of the herds [1]. In that work, the diagnostic power of saliva biomarkers to detect disease conditions in pigs was analysed in a multi-herd experimental approach, under field commercial conditions using two animal groups, healthy and diseased, and matching by breed, gender and age. The present study goes further on the full characterization of these analytes to ensure that saliva could be considered as an alternative specimen to serum for detection of disease in pigs. Furthermore, these results will help to find the best combination of biomarkers to develop an optimal algorithm, for its possible implementation for disease monitoring and/or health status assessment in the field, as suggested previously [17]. However, further studies should be performed to characterize the behaviour of each biomarker under different health conditions to define optimal analytical models for detection of homeostasis dysregulation’s in field conditions.

## Conclusions

The concentration of the studied salivary biomarkers of health and stress status showed variations during the daytime, so it is necessary to establish a time interval for routine saliva sampling for proper interpretation. The time interval between 10:00 a.m. and 12:00 a.m. is highly recommended since is compatible to both farm and laboratory labour. Sex and production stage influence the concentration of salivary biomarkers in healthy animals and both factors should be considered for reference range values calculation. We have established actual reference intervals for male and female pigs at post-weaning, growing and finishing states for 13 salivary biomarkers that will contribute to the use of saliva as a non-invasive sample for the diagnosis and monitoring of the health and stress status of swine farms.

## Materials and methods

### Aim, design and setting of the study

The aim of the study was to analyse the possible influence of different factors on the concentration of salivary biomarkers under healthy conditions that should be considered for proper clinical interpretation. Specifically, the factors to be studied were the circadian rhythm variations, and the possible differences according to sex and production phase of the pigs. To overcome the objective of the study, two separated trials were performed: circadian rhythm trial and RI trial.

For the first trial, a group of 40 animals around 120 days of life were sampled at different daytimes (07:00, 11:00, 15:00 and 19:00 h) on May 25th, 2022. The room temperature oscillated a maximum of 2.4ºC between samplings (from a minimum medium temperature of 22 ºC at early morning to a maximum medium temperature of 24.1 ºC in the afternoon). Four experimented veterinary researchers performed the clinical examination, to discard any clinical sign of disease, and the sampling of animals at each timepoint (1 researcher/10 animals) to reduce the duration of the experimental procedure.

The RI trial was performed in a total of 6 days between June 1st and 28th, 2022. Three different production phases were included in the study: post-weaning (pigs with a median weight of 21.6 kg), fattening (pigs with a median weight of 63.4 kg) and finishing (pigs with a median weight of 98.7 kg). Three farms per production phase were included in the study, in which a minimum of 20 males and 20 females were sampled at the same time of the day (between 10 and 12 h), after discarding any clinical signs of disease during proper clinical examination. A minimum of 120 animals (60 males and 60 females) were selected per production phase which gave a minimum total sample size of 360 pigs (Table 5).

**Table 5:** Characteristics of the animals from the reference interval analysis

Origin	N^1^	Age^2^	Breed	Weight^3^
Post-weaning				
Farm 1	Male 32	85	F1 Danbred x Duroc Danbred	22
	Female 31	85	F1 Danbred x Duroc Danbred	22
Farm 2	Male 23	63	F1 Danbred x Duroc Danbred	18
	Female 24	63	F1 Danbred x Duroc Danbred	18
Farm 3	Male 25	85	F1 Danbred x Duroc Danbred	25
	Female 25	85	F1 Danbred x Duroc Danbred	25
Growing				
Farm 4	Male 23	121	F1 ADN x Duroc Danish	59
	Female 22	120	F1 ADN x Duroc Danish	55
Farm 5	Male 21	120	F1 Danbred x Duroc Danbred	54
	Female 23	120	F1 Danbred x Duroc Danbred	54
Farm 6	Male 20	138	F1 Danbred x Duroc Danbred	79
	Female 22	147	F1 Danbred x Duroc Danbred	72
Finishing				
Farm 7	Male 21	170	F1 Danbred x Duroc Danbred	97
	Female 21	165	F1 Danbred x Duroc Danbred	89
Farm 8	Male 21	171	F1 Danbred x Duroc Danbred	103
	Female 21	165	F1 Danbred x Duroc Danbred	90
Farm 9	Male 20	194	F1 Danbred x Duroc Danbred	115
	Female 20	194	F1 Danbred x Duroc Danbred	115

^1^N = number of animals

^2^Age expressed as the mean value in days of life

^3^Weight expressed as the mean value in Kg

### Animals and housing conditions

For circadian rhythm trial, one commercial farm from the southeast of Spain was selected while for reference range interval trial, nine commercial farms from the Southeast of Spain were selected from the same commercial company to obtain data over different environments.

The vaccination of animals was the same for all farms within each production phase. The vaccination program consisted of the administration of a first dose against porcine circovirus and mycoplasma before weaning at 21 days of life, the vaccination against enzootic pneumonia at 10 and 14 weeks of life and the vaccination against Aujezsky disease at 11 and 15 weeks of life.

All pigs were housed in pen groups with 0.65 m2/pig following the official standards [54] with ad libitum access to balanced dry food and water.

The sample size used for the circadian rhythm trial was 40 (20 males and 20 females) as reported before [27]. To ensure a proper statistical significance of the analysis, the optimal power for daytime variations was established as 90%.

For the establishment of RIs, a minimal sample size of 60 per condition was used following the general guidelines for the determination of reference intervals in veterinary species [46].

## Saliva sampling procedure

Saliva samples were collected individually, without animal restrain, by using 1 × 1 × 1 cm sponges clipped to a thin metal rod. Pigs were allowed to chew the sponge for 1–2 min. Afterwards, sponges were included in specifically designed tubes for saliva collection (Salivette tubes, Sarstedt, Nümbrecht, Germany), individually labelled and stored in boxes with cold accumulators until transported to the laboratory within 4 h after collection. Saliva collection tubes were centrifuged at 3000 g for 10 min to obtain the clear whole saliva from the sponges and remove food or cell debris. Saliva samples were stored at -80ºC until analysis within 1 week.

### Acute phase proteins determination: CRP, Hp and Pig-MAP

The measurement of CRP and Hp was carried out using previously in-house developed time-resolved immunofluorometric assays (TR-IFMA) which were validated for the optimal quantification of CRP [55] and Hp [56] in porcine saliva samples. The concentrations of Pig-MAP were quantified using an in-house TR-IFMA, recently optimized and validated for proper saliva quantification from a previous ELISA [57]. In summary, the new developed assay showed good intra-assay and inter-assay precision with coefficient of variation (CV) lower than 10.56% and 13.73% respectively, good accuracy investigated by linearity under dilution with coefficient of correlation of 0.99 and a limit of detection of 4.09 ng/mL. The fluorometric signals were quantified in a multilabel counter (Victor 1420, Perkin Elmer, Turku, Finland). For saliva Pig-MAP determinations, saliva samples were analysed undiluted, and the calibration curve used covered a range between 7.8 and 2000 ng/mL.

### S100A12 quantifications

S100A12 levels were quantified by an in-house sandwich enzyme-linked immunosorbent assay (ELISA) recently developed and validated for porcine saliva determinations [58]. The assay consisted in a sandwich ELISA with good analytical parameters. In summary, the assay showed a high precision, with coefficients of variations lower than 7% for intra and inter-assay precision, good accuracy with coefficient of correlation of 0.98, when linearity under dilution was evaluated, and a limit of detection of 3.19 ng/mL. For saliva determinations, samples were diluted 1:1000 and the calibration curve used was constructed using porcine recombinant protein (1.95 to 125 ng/mL) (GenScript Biotech, Leiden, Netherlands).

### Measurement of Cu and Zn levels

For the measurements of Cu and Zn levels, samples were subjected to acid digestion as reported before [17] followed by atomic absorption spectrometry (Varian model SpectrAA 55B spectrometer, Palo Alto, CA, USA). The contents of Cu and Zn in the saliva samples were calculated by the interpolation of the signal in a standard curve (from 1 to 4 µg/mL of certified standard solutions for Cu and Zn (Agilent Technologies Spain, Madrid, Spain)), with standard concentration on the x-axis and signal on the y-axis drawing the best-fit curve through the standard points and taking into account the dilution of the saliva according to the digestion protocol used (0.5mL of saliva in a final volume of 2.5 mL).

### ADA activity determination

The procedure is an adaptation to microtitration plates of a commercial automatized assay (BioSystems S.A., Barcelona, Spain) based on Slaats et al. [59]. The assay was previously optimized and validated for porcine saliva samples with good precision, accuracy and limit of detection [14]. The levels of total ADA activity are calculated in U/mL according to manufacturer’s instructions taking into account the dilution factor.

### Total protein content determination

The total protein content was determined according to Bradford protocol [60]. Saliva samples were diluted (1:40) for its proper quantification using an albumin calibration curve (from 5 to 100 ng/mL).

### TAC, TOS and OSI determinations

TAC was measured through the ferric reducing antioxidant power (FRAP) assay [61]. The assay was validated for porcine saliva samples with good analytical performance [16]. The levels of TAC are calculated using a standard curve of Trolox (a water-soluble analogue of vitamin E employed as a control antioxidant agent for assay calibration), ranging from 1.5 to 100 µM Trolox equivalents/L.

The procedure for Total Oxidant Status (TOS) measurement is an in-house adaptation of a commercially available assay (Pierce Quantitative Peroxide Assay Kit, aqueous-compatible formulation) based on Nourooz-Zadeh et al. [62]. The assay has been previously validated for porcine saliva determinations with high precision, accuracy and good limit of detection [63]. The levels of TOS are calculated using a standard curve of Hydrogen peroxide (0.97 to 31.25 µM Peroxidase equivalents/L).

The Oxidative Stress Index (OSI) was calculated as the ratio TOS/TAC according to previous studies [64].

### Cortisol and salivary alpha-amylase measurements

The cortisol content was measured using an optimized commercial competitive ELISA (Extended range high sensitivity salivary cortisol Enzyme immunoassay kit, Salimetrics, USA). Cortisol concentration in µg/dL was calculated according to the manufacturer’s instructions by interpolation of the signal in a standard curve (0.012 µg/dL − 3 µg/dL) using porcine saliva without previous dilution.

Salivary alpha-amylase was quantified using an adaptation of the commercial kinetic enzyme assay (Salivary alpha-amylase kinetic enzyme assay kit, Salimetrics, USA). In the adaptation, 8 µL of saliva sample or control were incubated with 320 µL of amylase substrate during 5 min at 37ºC. The increase in absorbance from minute 1 to 5 was recorder for alpha-amylase activity calculation.

### Statistical analysis

Prior to statistical analysis, all groups of data were subjected to Shapiro-Wilk normality test followed by Fligner-Killeen test of homogeneity of variances to check the normality and homoscedasticity of the data and select the appropriate statistical tool. Data from both the circadian rhythm and RI trials passed the normality and homoscedasticity criteria so mixed ANOVA and two-way ANOVA test were used (for detailed results see supplementary Tables 1 and 2).

To show if differences exist between the timepoints during the daytime, results from the circadian rhythm trial were analysed by mixed ANOVA in which Maulchly’s test was used to check for sphericity using Greenhouse-Geisser or Huynh-Feldt sphericity corrections. When statistically significant differences were detected in the mixed ANOVA, a post-hoc multiple comparisons test was performed with Tukey’s correction and the size effect was calculated as the coefficient Cohen’s d. The achieved power for the mixed ANOVA test in the sample size studied was calculated for each biomarker. Afterwards, time series were analysed by the mixed-effects cosinor model to obtain a 24 h fitted cosine curve for each parameter and to estimate the effects of biological sex on each parameter curve (waveform). The estimated mean and confidence intervals for the means were calculated for the MESOR, amplitude and acrophase by sex and pairwise contrast by sex for each component were estimated [18, 25].

For the calculation of the RIs, histograms were prepared to illustrate the distribution of data and highlight possible outliers. Horn’s algorithm was used for outlier detection and elimination. Two-way ANOVA test were used considering sex and production phase as factors. When differences in the biomarker’s concentration due to sex or production phase were observed, partitioning criteria were used for more refined RI calculation within subgroups. A minimum of 120 reference animals were defined as the limit for the determination of reference limits by nonparametric methods. Bootstrapping was used to determine 90% confidence intervals for groups of less than 120 animals, following general recommendations [46].

All statistics were performed using R software version 4.0.3. The level of significance was set at p < 0.05.

### Electronic supplementary material

Below is the link to the electronic supplementary material.

Supplementary Material 1

## Data Availability

The datasets generated and analyzed during the current study are available in the Digitum repository, http://hdl.handle.net/10201/130522.

## Declarations

### List of abbreviations

ADA, Adenosine deaminase.

APPs, acute phase proteins.

CRP, C-reactive protein.

ELISA, Enzyme-linked immunosorbent assay.

Hp, Haptoglobin.

Ig, Immunoglobulins.

IL, Interleukin.

MESOR, Midline-estimating statistics of rhythm.

RI, Reference intervals.

TAC, Total antioxidant capacity.

TOS, Total oxidant status.

TP, Total protein.

TR-IFMA, Time-resolved immunofluorometric assay.

OSI, Oxidative stress index.
