# Supplementary material for: Influence of the circadian cycle, sex and production stage on the reference values of parameters related to stress and pathology in porcine saliva
Source: Porcine Health Manag. 2023 Sep 29;9:42. doi: 10.1186/s40813-023-00337-7 (PMC10541711; doi:10.1186/s40813-023-00337-7)
Supplement: Supplementary file 1 — Supplementary Material 1 [file 40813_2023_337_MOESM1_ESM.docx]

**Supplementary table 1.** Results of the statistical test performed to assess the normality distribution criteria and the homogeneity of variances of the different groups of data analyzed in the circadian rhythm trial.

| Biomarker | Shapiro-Wilk normality test for each group^1^ | Filgner-Killeen homogeneity of variances test^2^ |
| --- | --- | --- |
| Pig-MAP | Female:  W = 0.479, p = 2.368e-15  Male:  W = 0.603, p = 2.222e-13 | X^2^ = 1.176, df = 1, p = 0.2781 |
| S100A12 | Female:  W = 0.758, p = 4.57e-10  Male:  W = 0.312, p < 2.2e-16 | X^2^ = 15.319, df = 1, p = 9.078e-05 |
| ADA | Female:  W = 0.811, p = 1.028e-08  Male:  W = 0.891, p = 5.581e-06 | X^2^ = 5.056, df = 1, p = 0.02436 |
| TAC | Female:  W = 0.844, p = 1.026e-07  Male:  W = 0.836, p = 5.711e-08 | X^2^ = 5.611, df = 1, p = 0.01784 |
| TOS | Female:  W = 0.824, p = 4.387e-08  Male:  W = 0.766, p = 7.05e-10 | X^2^ = 2.066, df = 1, p = 0.1506 |
| OSI | Female:  W = 0.860, p = 6.575e-07  Male:  W = 0.471, p = 2.221e-15 | X^2^ = 1.258, df = 1, p = 0.262 |
| Cortisol | Female:  W = 0.907, p = 3.045e-05  Male:  W = 0.862, p = 4.337e-07 | X^2^ = 3.133, df = 1, p = 0.07669 |
| Amylase | Female:  W = 0.909, p = 3.265e-05  Male:  W = 0.888, p = 3.937e-06 | X^2^ = 4.162, df = 1, p = 0.04134 |
| CRP | Female:  W = 0.951, p = 0.005362  Male:  W = 0.923, p = 0.0001463 | X^2^ = 4.229, df= 1, p = 0.03972 |
| Hp | Female:  W = 0.688, p = 1.197e-11  Male:  W = 0. 704, p = 2.133e-11 | X^2^ = 17.629, df = 1, p = 2.685e-05 |
| TP | Female:  W = 0.974, p = 0.1209  Male:  W = 0.848, p = 1.49e-07 | X^2^ = 0.005, df = 1, p = 0.9402 |
| Zn | Female:  W = 0.938, p = 0.001824  Male:  W = 0.885, p = 5.266e-06 | X^2^ = 1.678, df = 1, p = 0.1952 |
| Cu | Female:  W = 0.854, p = 9.207e-07  Male:  W = 0.788, p = 4.296e-09 | X^2^ = 3.676, df = 1, p = 0.0552 |

^1^Statistic: W; level of significance: p value.

^2^Statistic: X^2^; degree of freedom: df; level of significance: p value.

**Supplementary table 2.** Results of the statistical test performed to assess the normality distribution criteria and the homogeneity of variances of the different groups of data analyzed in the reference interval trial.

| Biomarker | Shapiro-Wilk normality test for each group^1^ | Filgner-Killeen homogeneity of variances test for each group^2^ |
| --- | --- | --- |
| Pig-MAP | Female:  W = 0.709, p < 2.2e-16  Male:  W = 0.786, p = 1.203e-15  Finishing:  W = 0.742, p = 2.158e-13  Growing:  W = 0.738, p = 2.415e-13  Post-weaning:  W = 0.791, p = 2.248e-13 | Age:  X^2^ = 3.599, df = 1, p = 0.05779  Sex:  X^2^ = 18.112, df = 2, p-value = 0.0001167 |
| S100A12 | Female:  W = 0.926, p = 1.496e-08  Male:  W = 0.907, p = 7.235e-10  Finishing:  W = 0.912, p = 1.077e-06  Growing:  W = 0.924, p = 1.934e-06  Post-weaning:  W = 0.944, p = 9.906e-06 | Age:  X^2^ = 1.409, df = 1, p = 0.2351  Sex:  X^2^ = 23.285, df = 2, p = 8.787e-06 |
| ADA | Female:  W = 0.896, p = 1.36e-10  Male:  W = 0.848, p = 3.18e-13  Finishing:  W = 0.923, p = 3.929e-06  Growing:  W = 0.918, p = 8.813e-07  Post-weaning:  W = 0.933, p = 1.303e-06 | Age:  X^2^ = 13.077, df = 1, p = 0.000299  Sex:  X^2^ = 63.02, df = 2, p = 2.067e-14 |
| TAC | Female:  W = 0.836, p = 4.669e-14  Male:  W = 0.833, p = 5.864e-14  Finishing:  W = 0.833, p = 2.04e-10  Growing:  W = 0.842, p = 1.616e-10  Post-weaning:  W = 0.897, p = 5.022e-09 | Age:  X^2^ = 10.249, df = 1, p = 0.001367  Sex:  X^2^ = 25.357, df = 2, p = 3.118e-06 |
| TOS | Female:  W = 0.748, p < 2.2e-16  Male:  W = 0.748, p < 2.2e-16  Finishing:  W = 0.757, p = 7.343e-13  Growing:  W = 0.883, p = 1.844e-08  Post-weaning:  W = 0.649, p < 2.2e-16 | Age:  X^2^ = 16.049, df = 1, p = 6.173e-05  Sex:  X^2^ = 101.47, df = 2, p < 2.2e-16 |
| OSI | Female:  W = 0.814, p = 1.105e-14  Male:  W = 0.915, p = 3.62e-09  Finishing:  W = 0.892, p = 1.086e-07  Growing:  W = 0.876, p = 9.17e-09  Post-weaning:  W = 0.809, p = 6.373e-13 | Age:  X^2^ = 4.571, df = 1, p = 0.03251  Sex:  X^2^ = 7.882, df = 2, p = 0.01942 |
| Cortisol | Female:  W = 0.924, p = 1.452e-08  Male:  W = 0.905, p = 6.355e-10  Finishing:  W = 0.946, p = 0.0001574  Growing:  W = 0.926, p = 4.047e-06  Post-weaning:  W = 0.825, p = 2.461e-12 | Age:  X^2^ = 6.825, df = 1, p = 0.008987  Sex:  X^2^ = 31.717, df = 2, p = 1.296e-07 |
| Amylase | Female:  W = 0.817, p = 6.277e-15  Male:  W = 0.768, p < 2.2e-16  Finishing:  W = 0.657, p = 1.345e-15  Growing:  W = 0.844, p = 2.034e-10  Post-weaning:  W = 0.846, p = 1.12e-11 | Age:  X^2^ = 4.200, df = 1, p = 0.04042  Sex:  X^2^ = 73.596, df = 2, p < 2.2e-16 |
| CRP | Female:  W = 0.741, p < 2.2e-16  Male:  W = 0.782, p = 8.838e-16  Finishing:  W = 0.729, p = 9.331e-14  Growing:  W = 0.746, p = 1.248e-13  Post-weaning:  W = 0.774, p = 8.184e-14 | Age:  X^2^ = 0.774, df = 1, p = 8.184e-14  Sex:  X^2^ = 18.61, df = 2, p = 9.095e-05 |
| Hp | Female:  W = 0.865, p = 2.133e-12  Male:  W = 0. 756, p = 1.359e-11  Finishing:  W = 0.843, p = 5.277e-10  Growing:  W = 0.886, p = 2.164e-08  Post-weaning:  W = 0.863, p = 2.004e-10 | Age:  X^2^ = 3.334, df = 1, p = 0.06784  Sex:  X^2^ = 5.726, df = 2, p = 0.05709 |
| TP | Female:  W = 0.945, p = 5.489e-07  Male:  W = 0.944, p = 5.535e-07  Finishing:  W = 0.941, p = 4.779e-05  Growing:  W = 0.976, p = 0.02868  Post-weaning:  W = 0.952, p = 3.238e-05 | Age:  X^2^ = 2.126, df = 1, p = 0.1448  Sex:  X^2^ = 18.665, df = 2, p-value = 8.85e-05 |
| Zn | Female:  W = 0.901, p = 8.101e-10  Male:  W = 0.816, p = 6.37e-14  Finishing:  W = 0.881, p = 4.692e-08  Growing:  W = 0.900, p = 3.316e-07  Post-weaning:  W = 0.777, p = 2.363e-13 | Age:  X^2^ = 10.029, df = 1, p = 0.001541  Sex:  X^2^ = 9.498, df = 2, p = 0.008657 |
| Cu | Female:  W = 0.940, p = 1.091e-06  Male:  W = 0.923, p = 4.762e-08  Finishing:  W = 0.927, p = 2.264e-05  Growing:  W = 0.898, p = 6.107e-07  Post-weaning:  W = 0.928, p = 1.672e-06 | Age:  X^2^ = 6.075, df = 1, p = 0.01371  Sex:  X^2^ = 0.228, df = 2, p = 0.8918 |

^1^Statistic: W; level of significance: p value.

^2^Statistic: X^2^; degree of freedom: df; level of significance: p value.
